# The association of posttraumatic stress disorder and metabolic syndrome: a study of increased health risk in veterans

**DOI:** 10.1186/1741-7015-7-1

**Published:** 2009-01-09

**Authors:** Pia S Heppner, Eric F Crawford, Uzair A Haji, Niloofar Afari, Richard L Hauger, Boris A Dashevsky, Paul S Horn, Sarah E Nunnink, Dewleen G Baker

**Affiliations:** 1 Veterans Affairs San Diego Health Care System, Research Service, MC 151, La Jolla Village Drive, San Diego, CA 92161, USA; 2 Department of Psychiatry, University of California at San Diego, Gilman Drive, MC:0603, La Jolla, CA 92093-0603, USA; 3 Durham Veterans Affairs Medical Center, Felton Street, Durham, NC 27705, USA; 4 Psychiatry Service, Cincinnati Veterans Affairs Medical Center, Vine Street, Cincinnati, OH 45220, USA; 5 Department of Mathematical Sciences, University of Cincinnati, Old Chemistry Building, Cincinnati, OH 45221-0025, USA

## Abstract

**Background:**

There is accumulating evidence for a link between trauma exposure, posttraumatic stress disorder (PTSD) and diminished health status. To assess PTSD-related biological burden, we measured biological factors that comprise metabolic syndrome, an important established predictor of morbidity and mortality, as a correlate of long-term health risk in PTSD.

**Methods:**

We analyzed clinical data from 253 male and female veterans, corresponding to five factors linked to metabolic syndrome (systolic and diastolic blood pressure, waist-to-hip ratio and fasting measures of high-density lipoprotein (HDL) cholesterol, serum triglycerides and plasma glucose concentration). Clinical cut-offs were defined for each biological parameter based on recommendations from the World Health Organization and the National Cholesterol Education Program. Controlling for relevant variables including sociodemographic variables, alcohol/substance/nicotine use and depression, we examined the impact of PTSD on metabolic syndrome using a logistic regression model.

**Results:**

Two-fifths (40%) of the sample met criteria for metabolic syndrome. Of those with PTSD (*n *= 139), 43% met criteria for metabolic syndrome. The model predicted metabolic syndrome well (-2 log likelihood = 316.650, chi-squared = 23.731, *p *= 0.005). Veterans with higher severity of PTSD were more likely to meet diagnostic criteria for metabolic syndrome (Wald = 4.76, *p *= 0.03).

**Conclusion:**

These findings provide preliminary evidence linking higher severity of PTSD with risk factors for diminished health and increased morbidity, as represented by metabolic syndrome.

## Background

Several lines of research suggest that stress and post-stress adaptational responses are related to long-term health outcomes. Studies of survivors of disasters, veterans and prisoners of war, and others exposed to severe trauma, suggest higher rates of physical morbidity and mortality and increased healthcare utilization related to lifetime prevalence of trauma [1-7]. There is accumulating evidence from epidemiological studies that chronic posttraumatic stress disorder (PTSD) may moderate the link between trauma and secondary negative health outcomes such as cardiovascular, metabolic and autoimmune conditions [4-9]. Furthermore, Chrousos and colleagues have linked maladaptive neuro-endocrine-immune responses to psychiatric, endocrine and/or autoimmune disease or vulnerability to such diseases [10,11]. Evidence for neuro-endocrine-immune abnormalities associated with PTSD, also provides a biological rationale for downstream health effects observed in the epidemiological studies [12-16].

Metabolic syndrome or syndrome × is composed of a cluster of clinical signs including dyslipidemia, hyperglycemia (insulin resistance), central obesity and hypertension [17]. Recent research on the impact of stress on health has focused on metabolic syndrome as a possible consequence of pathophysiological adaptations to chronic stress [18]. Brunner and colleagues studied metabolic syndrome status in the Whitehall II cohort and found markers associated with increased stress-related neuroendocrine and autonomic activation such as lowered heart rate variability, increased cortisol output and higher levels of IL-6, C-reactive protein and blood viscosity among cases with metabolic syndrome as compared with those who did not meet diagnostic criteria [19]. Additional analyses of prospective data from the Whitehall cohort also suggest a dose-response relationship between stress and the presence of metabolic syndrome such that participants with chronic exposure to work stress were found to be more than twice as likely to have the syndrome after controlling for age, sex and health behaviors [20]. In a recent study of 2189 Gulf War I veterans, those with chronic multisymptom illness, as defined by presence of fatigue, musculoskeletal pain, mood or cognitive abnormalities for at least 6 months, had significantly higher prevalence of metabolic syndrome [21].

While there is a lack of consensus in specific clinical definitions of metabolic syndrome, prospective studies have shown it to be an important predictor of physical morbidity and mortality [22-25]. For example, in a comparison of four definitions based on guidelines from the World Health Organization (WHO) and the National Cholesterol Education Program (NCEP), metabolic syndrome was found to predict incidence and prevalence of diabetes mellitus in a prospective study [24]. Similarly, in a sample of 5128 men, metabolic syndrome was found to be a significant predictor of the development of cardiovascular disease (CVD) and diabetes mellitus [25].

In this study, we examined physical measures and laboratory values in a sample of veterans, to examine the association between PTSD severity and the presence of metabolic syndrome. We hypothesized that greater severity of PTSD would be associated with a higher likelihood of metabolic syndrome.

## Methods

### Participants

Comprehensive medical and mental health examinations were completed by 355 veterans entering Gulf War Screening and PTSD Programs at the Cincinnati Veterans Affairs, of whom 341 signed informed consents permitting use of their data for a health-related study. The study was conducted in accordance with the Helsinki Declaration, and was approved by the Institutional Review Board of the University of Cincinnati Medical Center and the Research Committee of the Cincinnati Veterans Affairs Medical Center (Protocol #00-03-07-01-E; "Health-related quality of life in post-deployment veterans").

Of the 341 veterans with signed consent, we excluded 68 veterans who had missing data from at least one of the variables of interest, 18 veterans whose laboratory values were greater than 3 standard deviations (SD) from the group mean, and 2 veterans who had both missing data and laboratory values greater than 3 SD from the mean. Cases with extreme laboratory values were excluded to allow for a more conservative analysis of quantitative physiological burden. These exclusions yielded a remaining sample size of 253 veterans.

### Measures

#### Sociodemographics

Veterans provided sociodemographic information including years of education, military service and deployment history via written questionnaires.

#### Psychiatric diagnoses

PTSD severity was measured using the Clinician Administered PTSD Scale (CAPS), which ranges in scores from 0 to 136 (see [26]). A score of 65 or above on the CAPS has been shown to be optimally specific and efficient in predicting PTSD and was used, along with the *Diagnostic and Statistical Manual of Mental Disorders, 4th Edition *(DSM-IV) criteria, to identify veterans with PTSD [27]. Lifetime and current diagnoses of major depressive disorder (MDD), substance, alcohol and nicotine abuse or dependence were determined using a structured diagnostic interview [28,29].

#### Physical measures

Physical examinations including waist-to-hip ratio (WHR), blood pressure and laboratory tests (12-hour fasting lipids, glucose) were conducted on each veteran. Phlebotomy was scheduled between 7 and 9 AM. All samples were assayed within the hospital clinical laboratory.

### Procedure

In the context of a cross-sectional study, metabolic syndrome status was determined based on the clinical criteria for each factor outlined in Table 1. Systolic and diastolic blood pressure values were combined to represent one condition (hypertension as represented by systolic blood pressure (SBP) ≥ 130 mm/Hg and diastolic blood pressure (DBP) ≥ 85 mm/Hg) yielding a total of five criteria that contributed to metabolic syndrome. While the cut-off for elevated serum triglycerides was based on NCEP criteria, increased blood pressure, high-density lipoprotein (HDL), WHR and plasma glucose concentration were based on a modified set of criterion scores recommended by the WHO and the NCEP, similar to those used by Lakka and colleagues to evaluate CVD-related mortality and metabolic syndrome [23,30-32].

**Table 1:** Clinically determined criterion scores for metabolic syndrome

**Measures**	**Criterion score**
Serum triglycerides	≥ 150 mg dl^-1^
High-density lipoprotein	< 35 mg dl^-1^
Blood pressure^a^	
Systolic	≥ 130 mm/Hg
Diastolic	≥ 85 mm/Hg
Waist-to-hip ratio	
Men	≥ 0.90
Women	≥ 0.85
Plasma glucose concentration	≥ 110 mg dl^-1^

^a^Systolic and diastolic blood pressure values were combined to represent one condition (hypertension as represented by SBP ≥ 130 mm/Hg and DBP ≥ 85 mm/Hg).

Metabolic syndrome status was defined as meeting of three or more of the five criteria.

### Statistical analyses

We examined the relationship between PTSD and metabolic syndrome using a logistic regression model. In addition, we conducted a series of independent-sample *t*-tests to determine whether those with PTSD differed on these five criteria from those without PTSD. Statistical analyses were conducted using SPSS (Statistical Package for Social Sciences) version 15.0. Metabolic syndrome was regressed on the following predictors: PTSD severity (CAPS), age, gender, race (white, black/other), years of education, history of substance abuse/dependence, history of alcohol abuse/dependence, nicotine abuse/dependence (lifetime) and current or past diagnosis of MDD. All predictors were simultaneously entered into the regression model as a single block in order to determine whether PTSD severity was a valid predictor of metabolic syndrome status, while controlling for all other demographic, behavioral and psychiatric variables.

## Results

### Participants

Table 2 describes the sociodemographic, medical and psychiatric characteristics of participants. The sample was primarily male and white, with an average age of 52 ± 9 years. A large proportion served in the US Army and more than 70% served in Vietnam; 55% of the veterans met criteria for PTSD, as defined by a CAPS score of more than 65 and all met DSM-IV criteria for PTSD based on the presence of intrusion, avoidance and hyperarousal scores. There were 54 veterans (21%) who did not meet criteria for PTSD based on the three DSM-IV symptom clusters and another 60 veterans (24%) who had subthreshold PTSD (reported all three types of symptom, but had a total CAPS score less than 65). Close to two-thirds of the participants met criteria for MDD. Abuse or dependence of nicotine, alcohol and other substances were also prevalent in this sample. In addition, there was high comorbidity between PTSD and MDD, lifetime nicotine abuse or dependence, past substance abuse or dependence, and past alcohol abuse or dependence.

**Table 2:** Sociodemographic, medical and psychiatric characteristics of veterans (*N *= 253)

**Sociodemographic data**	**Number (percentage) of all veterans**
Male	233 (92%)
Race	
White	193 (76.3%)
Black	47 (18.6%)
Other	13 (5.1%)
Branch of service	
Army	153 (60.5%)
Marines	53 (20.9%)
Navy	26 (10.3%)
Air Force	21 (8.3%)
Service era	
World War II	7 (2.8%)
Korea	8 (3.2%)
Vietnam	180 (71.1%)
Gulf War I	36 (14.2%)
Other conflicts	22 (8.7%)
Medical and psychiatric diagnoses	
PTSD	
At least moderate severity	139 (54.9%)^a^
Subthreshold severity	60 (24.0%)^a^
MDD	163 (64.4%)
Nicotine abuse/dependence (lifetime)	98 (38.7%)
Substance abuse/dependence (past)	102 (40.3%)
Alcohol abuse/dependence (past)	174 (68.8%)
Co-morbid PTSD and MDD	104 (41.1%)^b^
Co-morbid PTSD and lifetime nicotine abuse/dependence	53 (20.9%)
Co-morbid PTSD and past substance abuse/dependence	67 (26.5%)
Co-morbid PTSD and past alcohol abuse/dependence	107 (42.3%)
Metabolic syndrome (prevalence)	101 (39.9%)^c^
PTSD only and metabolic syndrome	12 (34.3%)^d^
MDD only and metabolic syndrome	17 (28.8%)^e^
PTSD, MDD and metabolic syndrome	48 (46.2%)^f^
**Other factors:**	
Age (mean ± standard deviation (SD)) = 51.5 ± 9.0 years	
Length of education (mean ± SD) = 12.9 ± 2.4 years	
CAPS score (mean ± SD) = 62.8 ± 29.4 years.	

^a^Those identified as meeting *Diagnostic and Statistical Manual of Mental Disorders, 4th Edition *(DSM-IV) criteria for posttraumatic stress disorder (PTSD) were subclassified into subthreshold (Clinician Administered PTSD Scale (CAPS) < 65) or moderate to severe PTSD (CAPS ≥ 65).

^b^Diagnosis of PTSD if CAPS ≥ 65; diagnosis of major depressive disorder (MDD) determined by structured psychodiagnostic clinical interview.

^c^Metabolic syndrome status if three of five criteria are present (serum triglycerides at least 150 mg dl^-1^, high-density lipoprotein less than 35 mg dl^-1^, blood pressure at least 130/85 mm/Hg, waist-to-hip ratio at least 0.90 for men and at least 0.85 for women, and plasma glucose concentration at least 110 mg dl^-1^).

^d^Percentage of those with PTSD only who meet criteria for metabolic syndrome.

^e^Percentage of those with MDD only who meet criteria for metabolic syndrome.

^f^Percentage of those with both PTSD and MDD who meet criteria for metabolic syndrome.

The overall prevalence of current metabolic syndrome in our sample was 39.9%. Among those with PTSD only, 34.3% (*n *= 12) met criteria for metabolic syndrome as compared with 28.8% (*n *= 17) of those with MDD only. For those with PTSD and MDD, 46.2% (*n *= 48) met criteria for metabolic syndrome. Mean values for physical and laboratory measures, shown in Table 3, indicate that, on average, the sample had elevated serum triglycerides, high total cholesterol/HDL ratios and central obesity. Group differences between those with and without PTSD on these physical and laboratory measures were not significant except for those with PTSD had significantly higher DBP measures than those without PTSD (*t *= -2.15, *p *= 0.033). These multiple *t*-tests evaluating group differences were not corrected for possible inflation of Type I error (that is, the Bonferroni principle).

**Table 3:** Physical measures and laboratory values used to determine metabolic syndrome

**Measures**	**All Veterans (*N *= 253)**	**With PTSD (*N *= 139)**	**Without PTSD (*N *= 114)**	***p***
Serum triglycerides	189.5 ± 141.8	194.6 ± 154.7	183.3 ± 124.7	ns
High-density lipoprotein	42.7 ± 11.3	42.5 ± 10.6	43.0 ± 12.0	ns
Blood pressure				
Systolic	130.8 ± 15.3	132.2 ± 16.0	129.1 ± 14.4	ns
Diastolic	81.7 ± 10.0	82.9 ± 9.9	80.2 ± 10.0	0.033
Waist-to-hip ratio				
Men	0.97 ± 0.07	0.98 ± 0.07	0.97 ± 0.06	ns
Women	0.85 ± 0.06	0.87 ± 0.03	0.84 ± 0.06	ns
Plasma glucose concentration	106.4 ± 26.8	105.6 ± 26.3	107.5 ± 27.5	ns

All values are mean ± standard deviation. Diagnosis of posttraumatic stress disorder (PTSD) if Clinician Administered PTSD Scale (CAPS) ≥ 65 and *Diagnostic and Statistical Manual of Mental Disorders, 4th Edition *(DSM-IV) criteria for PTSD are met. ns, not significant.

### PTSD and metabolic syndrome

Table 4 presents the results of the logistic regression model used to predict presence of metabolic syndrome. The overall regression model produced an adequate model fit (-2 log likelihood = 316.650, chi-squared = 23.731, *p *= 0.005). CAPS total score (Wald = 4.76, *p *= 0.03, odds ratio (OR) = 1.01) was a significant predictor of metabolic syndrome. Regression coefficients indicated that veterans' risk for metabolic syndrome increased one percentage point for each point obtained on the CAPS. Gender was also a significant, unique predictor of risk for metabolic syndrome with women having lower risk (Wald = 4.852, *p *= 0.028, OR = 0.17). To determine whether the comorbidity between PTSD and MDD in our sample (41%) would introduce problems related to multicollinearity to our model, we conducted diagnostic tests (variance inflation factor (VIF) estimates and tolerance) and found no evidence for multicollinearity (VIF = 1.053, tolerance = 0.950).

**Table 4:** Logistic regression predicting metabolic syndrome score

**Variable**	**Estimate**	**Standard error**	**Wald**	***P***	**OR**	**95% CI**	
						**Lower**	**Upper**
Age	0.032	0.017	3.710	0.054	1.033	0.999	1.068
Race	-0.329	0.324	1.031	0.310	0.719	0.381	1.358
Gender	-1.772	0.805	4.852	0.028	0.170	0.035	0.823
Years of education	0.013	0.059	0.051	0.821	1.014	0.903	1.138
Nicotine abuse	-0.439	0.281	2.441	0.118	0.645	0.372	1.118
Substance abuse	0.418	0.294	2.015	0.156	1.519	0.853	2.704
Alcohol abuse	-0.317	0.332	0.915	0.339	0.728	0.380	1.395
MDD	-0.274	0.293	0.876	0.349	0.760	0.429	1.349
CAPS	0.011	0.005	4.759	0.029	1.011	1.001	1.022

'Race' reflects the difference in metabolic risk comparing veterans of non-white to those of white ethnicity. MDD, major depressive disorder. 'CAPS' reflects one unit change in metabolic risk associated with one unit change in Clinician Administered PTSD Scale (CAPS) score.

## Discussion

In a sample of 253 male and female veterans, greater severity of PTSD was associated with higher likelihood of metabolic syndrome after controlling for relevant demographic, behavioral and psychiatric factors. This finding aligns with and extends existing research examining metabolic syndrome in populations vulnerable to PTSD. Violanti and colleagues found metabolic syndrome to be three times more likely in police officers with severe PTSD symptoms as compared with police officers in the lowest PTSD severity category [33], while others have found that that 31–35% of male war veteran samples with combat PTSD evidenced concomitant metabolic syndrome [34,35]. Of note, the current study utilized the largest sample size to date and the most diverse in terms of gender, given the small but extant presence of women.

The importance of the association between PTSD and metabolic syndrome becomes apparent when one considers the fact that metabolic syndrome has been shown to predict CVD-related morbidity and mortality [23,25]. Our findings suggest that metabolic syndrome provides a useful framework for assessing and describing the physiologic burden of PTSD and can be used prospectively to evaluate the increased health risk associated with combat exposure and PTSD.

The prevalence of metabolic syndrome in our sample (40%) and specifically among those with PTSD (43%) were slightly higher than rates observed among adults in the general population as reported in the National Health and Nutrition Examination Survey (NHANES) which ranged from 20% to 31% [36-38]. Loucks and colleagues reported mean values from men and women ages 25 years and over on individual metabolic syndrome components also collected in our sample (HDL, serum triglycerides, SBP, DBP, and fasting glucose) [36]. Compared with the women and men surveyed in NHANES, our sample of veterans tended to have higher mean triglycerides (189.5 mg dl^-1 ^versus 132.3 and 164.0 mg dl^-1 ^for women and men, respectively), higher SBP (130.8 mm/Hg versus 122.1 and 123.1 mm/Hg), higher DBP (81.7 mm/Hg versus 71.2 and 74.4 mm/Hg), higher fasting glucose (106.4 mg dl^-1 ^versus 98.8 and 103.4 mg dl^-1^), and lower HDL (42.7 mg dl^-1 ^versus 55.8 and 45.3 mg dl^-1^). While our sample appeared to be 'less healthy' based on the pattern of mean values, our participants also tended to be older (mean age of 51.5 years as compared with mean ages of 47.2 and 44.7 years for women and men, respectively, in NHANES).

In spite of the growing attention on the importance and surveillance of metabolic syndrome, it should be noted that there is still considerable controversy regarding its definition and utility. Recent reviews pose questions regarding the need for a consensus in the definition of metabolic syndrome, lack of certainty about the underlying pathophysiological process, whether the syndrome itself conveys more information about health risk than the sum of its individual components, and how different combinations of risk factors should guide clinical management [39-41]. In addition to research examining long-term health risk from PTSD, prospective studies are similarly needed to continue to address these remaining questions about metabolic syndrome.

A closely related concept, allostatic load, represents the cumulative biological burden of exposure to repeated cycles of adaptation to stress [42]. It incorporates the notion of stress, as well as the contribution of genetic, developmental, and lifestyle factors that might underpin the physiologic response to daily life events and traumatic stressors [42-45]. The selected factors that contribute to metabolic syndrome in this study closely approximate the secondary mediators of allostatic load, with the exception of serum triglycerides [46,47]. Our cross-sectional findings show a relationship between PTSD and metabolic syndrome suggesting that perhaps metabolic syndrome in itself is a useful clinical tool in quantifying the cardiovascular and metabolic impact of PTSD. In comparing those with and without PTSD on individual measures of metabolic syndrome components, we only found significant group differences for DBP, suggesting the utility of considering these measures collectively as the presence or absence of metabolic syndrome. It should be noted, however, that these multiple comparisons were not adjusted according to the Bonferroni principle, thereby potentially inflating Type I error in our analysis. Several studies appear to support the dysregulating effect of PTSD on a number of physiologic systems [12-16,33-35,48]. Given this association, future prospective studies examining physical morbidity and mortality related to PTSD should also assess metabolic syndrome to determine whether the pattern of dysregulation represented by metabolic syndrome mediates long-term health risk in PTSD.

Interestingly, we did not find MDD to be a significant and unique predictor of risk for metabolic syndrome, in spite of preliminary findings suggesting increased HPA activity with depression [49-55]. These previous studies, however, focused on depression and did not assess or control for PTSD. Our findings may provide preliminary evidence for a higher relative contribution of PTSD to metabolic syndrome after controlling for MDD.

One limitation of the current study is the under-representation of women and minority groups. While female gender was found to be associated with decreased risk of metabolic syndrome, in spite of the small number of women in the study (7.9%), race did not appear to have unique effects on metabolic syndrome. A more diverse sample may yield important information on whether the relationship between PTSD and metabolic syndrome is robust across different racial/ethnic groups.

Finally, given the cross-sectional design of our study, we cannot fully establish a causal relationship between PTSD and metabolic syndrome. Prospective studies are clearly needed in this area to fully examine the long-term health risk related to PTSD.

## Conclusion

This study extends the current knowledge of PTSD and diminished health status by examining metabolic syndrome while controlling for relevant health and psychiatric variables. After controlling for MDD, and alcohol, nicotine and substance abuse or dependence, we found a robust and significant association between PTSD severity and health risk as measured by metabolic syndrome. Further exploration, using prospective studies with assessment of CVD- and metabolic-related conditions, are indicated to determine whether metabolic syndrome can sufficiently account for the higher morbidity and mortality observed among those exposed to trauma or with PTSD.

## Abbreviations

CAPS: Clinician Administered PTSD Scale; CVD: cardiovascular disease; DBP: diastolic blood pressure; DSM-IV: *Diagnostic and Statistical Manual of Mental Disorders, 4th Edition*; HDL: high-density lipoprotein; HPA: hypothalamic-pituitary-adrenocortical axis; MDD: major depressive disorder; NCEP: National Cholesterol Education Program; NHANES: National Health and Nutrition Examination Survey; OR: odds ratio; PTSD: posttraumatic stress disorder; SBP: systolic blood pressure; SD: standard deviations; SPSS: Statistical Package for Social Sciences; VIF: variance inflation factor; WHO: World Health Organization; WHR: waist-to-hip ratio.

## Competing interests

The authors declare that they have no competing interests.

## Authors' contributions

PSH (1) conducted statistical analyses and participated in drafting the manuscript. EFC was involved with statistical analysis and writing the manuscript. UAH was involved in data collection and study execution. NA participated in interpretation of the findings, as well as manuscript drafting and editing. RLH participated in editing the manuscript. BAD was involved with data processing and analysis and database construction. PSH (2) participated in statistical analyses. SEN assisted with manuscript editing and formatting. DGB carried out study design, data gathering and participated in manuscript drafting and editing.

## Pre-publication history

The pre-publication history for this paper can be accessed here:


